# Psychological Impact of COVID-19 on Parents of Pediatric Cancer Patients

**DOI:** 10.3389/fpsyg.2021.730341

**Published:** 2021-09-22

**Authors:** Antonella Guido, Elisa Marconi, Laura Peruzzi, Nicola Dinapoli, Gianpiero Tamburrini, Giorgio Attinà, Mario Balducci, Vincenzo Valentini, Antonio Ruggiero, Daniela Pia Rosaria Chieffo

**Affiliations:** ^1^UOS Psicologia Clinica, Fondazione Policlinico Universitario A. Gemelli IRCCS, Università Cattolica Sacro Cuore, Rome, Italy; ^2^Pediatric Oncology Unit, Fondazione Policlinico Universitario A. Gemelli IRCCS, Università Cattolica Sacro Cuore, Rome, Italy; ^3^UOC Radioterapia Oncologica, Dipartimento Diagnostica per Immagini, Radioterapia Oncologica ed Ematologia, Fondazione Policlinico Universitario A. Gemelli IRCCS, Rome, Italy; ^4^Pediatric Neurosurgery, Fondazione Policlinico Universitario A. Gemelli IRCCS, Università Cattolica del Sacro Cuore, Rome, Italy

**Keywords:** psycho-oncology, pediatric oncology, COVID-19, parent perception, cancer, children, stress, quality of life

## Abstract

The changes and general alarm of the current COVID-19 pandemic have amplified the sense of precariousness and vulnerability for family members who, in addition to the emotional trauma of the cancer diagnosis, add the distress and fear of the risks associated with infection. The primary objectives of the present study were to investigate the psychological impact of the COVID-19 pandemic on the parents of pediatric cancer patients, and the level of stress, anxiety, and the child’s quality of life perceived by the parents during the COVID-19 epidemic. The parents of 45 consecutive children with solid and hematological tumors were enrolled. Four questionnaires (Impact of Event Scale-Revised – IES-R; Perceived Stress Scale – PSS; Spielberger State – Trait Anxiety Inventory – STAI-Y; Pediatric Quality of Life Inventory – PedsQL) were administered to the parents at the beginning of the pandemic lockdown. A 75% of parents exhibited remarkable levels of anxiety, with 60 subjects in state scale and 45 subjects in trait scale having scores that reached and exceeded the STAI-Y cut off. The bivariate matrix of correlation found a significant positive correlation between the IES-R and PSS scores (*r* = 0.55, *P* < 0.001). There was a positive correlation between the PSS and PedsQL (emotional needs) scale (*P* < 0.001) and a negative correlation between IES-R and STAI-Y (*P* < 0.001). The results confirm that parents of pediatric cancer patients have a high psychological risk for post-traumatic symptoms, high stress levels, and the presence of clinically significant levels of anxiety.

## Introduction

The COVID-19 pandemic has affected several aspects of lives all around the globe, and the unprecedented health crisis has put a strain on health services. The literature shows that lockdown measures can affect mental health with several psychological consequences: anxiety, stress, depression, frustration, irritability, insomnia, post-traumatic stress symptoms, and anger (Brooks et al., 2020; Di Giuseppe et al., 2020; Franceschini et al., 2020; Osimo et al., 2021). In Italy and Spain children show increasing screen time, less physical activity, and more sleep; many parents reported changes in their children’s behavior and emotional state (Ferrari et al., 2020; Orgilés et al., 2020) and higher levels of parental burnout were reported, especially parents of children with a mental or physical disorder (Fontanesi et al., 2020).

Cancer patients were particularly affected, due to their vulnerability, immunosuppression, or need for cancer treatment (Tsamakis et al., 2020) resulting in a high psychological impact (Jones et al., 2020). Among oncology patients, infants and children are at higher risk for medical or psychological complications (Bitsko et al., 2016; Brinkman et al., 2018). The psycho-evolutionary implications of antineoplastic treatments are well known (Moore, 2005; Oppenheim, 2007; Miller et al., 2009; Brand et al., 2017; Stavinoha et al., 2018), in fact pediatric cancer patient is exposed to continuous events over time that can fall within the field of traumatic stress (Bertolotti et al., 2017). Clinical experience in pediatric oncology shows that trauma can cause psychopathological conditions in survivors (Stuber et al., 2010; Clerici et al., 2014) and also described in the literature (Axia, 2004; Guarino, 2006; Bertolotti and Massaglia, 2011; Hildenbrand et al., 2011), framing pediatric cancer as a stressful and traumatic life cycle event (Patenaude and Kupst, 2005; Phipps et al., 2005; Currier et al., 2009).

The COVID-19 epidemic can represent a further stressful event that is part of a vulnerability framework of the pediatric cancer patient, constituting an additional psycho-pathological risk factor. The researchers on severe psychological trauma (Liotti, 2004; Fosha et al., 2009; Nijenhuis and van der Hart, 2011) and child trauma expert (Lanius et al., 2010, 2012) describe “complex trauma” (Van Der Kolk, 1996; Cook et al., 2005) such as experience of multiple, chronic and prolonged traumatic events (Van der Kolk, 2005).

The risk of COVID-19 infection, and the unpredictability of relative potential emergencies, could exacerbate the emotional burden on patients and family members during oncological disease and treatment. The changes and general alarm of the current pandemic have amplified the sense of precariousness and vulnerability for family members who, in addition to the emotional trauma of the cancer diagnosis, add the distress and fear of the risks associated with infection. The parents fear the consequences of infection on their child’s already fragile state of health as well as potential treatment interruptions or delays.

In addition to the standard complex oncological clinical pathway, they require additional measures of self-protection, social distancing (André et al., 2020), prolonged isolation, and new daily habits (Clerici et al., 2020). Also, hospital rules have become more restrictive, requiring the suspension of some services and limitations to family visitation (Leung et al., 2020). These factors significantly affect the patients and their family’s quality of life both during hospitalization and afterward upon discharge.

The primary objectives of the present study was to investigate the psychological impact of the COVID-19 pandemic on the parents of pediatric cancer patients, and to investigate the level of stress, anxiety, and the child’s quality of life perceived by the parents during the COVID-19 epidemic. Subsidiary objective of the study was to explore correlations between the results obtained and the variables investigated.

## Materials and Methods

Our study is a single center prospective observational study; duration 9 months. Parents of pediatric cancer patients were enrolled during the 3 months, June–August 2020. Subsequently, the sample was distributed in two groups: parents of patients in treatment (GT) and parents of patients in off-therapy (GOT). Data from the literature report that the level of anxiety and distress of parents, very hight after the diagnosis of their child, can be reduced already during the first 3 months by up to 66% (Harper et al., 2013; Scarponi et al., 2017). Considering the hypothesis of mild correlation (*r* = 0.3) between Impact of Event Scale-Revised and Perceived Stress Scale, an alpha error = 0.05 (two tailed, probability for rejecting the null hypothesis, type I error rate), and a beta error = 0.20 (probability of failing to reject the null hypothesis under the alternative hypothesis, type II error rate) the calculated sample size was 85 cases (Hulley et al., 2013). We concluded the recruitment of the subjects before the expected number of parents was obtained because the recruitment period had ended. Nevertheless, the results obtained confirm the hypothesis of the study.

### Participants

The parents of 45 consecutive children with solid and hematological tumors treated in the Pediatric Oncology, Pediatric Neurosurgery, and Radiotherapy Units of Fondazione Policlinico Universitario A. Gemelli IRCCS in Rome were enrolled in the study. Criteria for selecting the subjects were: (1) parent of a patient with a cancer diagnosis; (2) parent of a patient who was in treatment or had completed their treatment regimen; and (3) parent of patients <25 years of age. The patients ≤25 years of age recruited in the study are those who belong to the Unit as suffering from pediatric cancer in treatment or follow-up. Parents with psychiatric or cognitive disorders or intellectual disability were excluded from the study. The parents recruited in the study were screened at the Psychology Service. Parents who were diagnosed with psychiatric disorder were excluded from the study.

This study was performed in accordance with the Helsinki declaration and approved by the Institutional Review Board. Written informed consent was obtained from all participants.

### Measures

#### Impact of Event Scale-Revised (IES-R)

Impact of Event Scale-Revised (IES-R) is a 22-item, self-report measure (for DSM-IV) that assesses subjective distress caused by traumatic events (Weiss and Marmar, 1997; Weiss et al., 2007). The IES-R measures distress, with three subscales assessing Avoidance, Intrusion, and Hyperarousal. In addition to the three subscale scores, IES-R also gives an overall score of events impact (IES-R total, equal to the sum of the three subscale scores). The cut-off of 33 was adopted to indicate a high risk of PTSD symptomatology, in line with the literature. The Italian translation of the IES – R showed satisfactory internal consistency in studies on different at-risk populations (Intrusion, α = 0.78; Avoidance, α = 0.72; Hyperarousal, α = 0.83) (Craparo et al., 2013; Forte et al., 2020). The IES-R is very helpful in measuring the effect of distress, and traumas in oncology (Nakajima-Yamaguchi et al., 2016).

#### Perceived Stress Scale (PSS)

Perceived Stress Scale (PSS) is a psychological instrument for measuring the perception of stress. The questions ask about feelings and thoughts during the previous few months (Mondo et al., 2019; Cusinato et al., 2020). The PSS-10 is a self-report instrument consisting of 10 items. Each of the items on the PSS-10 are rated on a 5-point Likert scale, ranging from 0 (never) to 4 (very often). The PSS-10 consisted of 6 positively (items 1, 2, 3, 6, 9, and 10: Positive factor) and 4 negatively (items 4, 5, 7, and 8: Negative factor) worded items. Total scores range from 0 to 40, with higher scores indicating higher levels of perceived stress. Scores ranging from 0 to 13 would be considered low stress. Scores ranging from 14 to 26 would be considered moderate stress. Scores ranging from 27 to 40 would be considered high perceived stress. It was frequently used during the pandemic in Italy and other countries (Limcaoco et al., 2020; Rossi et al., 2020). Internal consistency estimates using Cronbach’s alpha range from 0.67 to 0.91.

#### Spielberger State – Trait Anxiety Inventory (STAI-Y)

Spielberger State – Trait Anxiety Inventory (STAI-Y) is a 40-item, self-completed questionnaire that aims to separately assess state anxiety (STAI-Y1, a temporary state influenced by the current situation) and trait anxiety (STAI-Y2, a general propensity to be anxious) with 20 items each (Pedrabissi and Santinello, 1989; Cafisio and Tralongo, 2004). Scores over 40 on both the state and trait scales were adopted; this value corresponded to the point at which false positive and negative results were minimal (Barnett and Parker, 1986; Hart and McMahon, 2006). The internal consistency reliability ranges from 0.91 to 0.95 for the scale of state and from 0.85 to 0.90 for the scale of trait.

#### Pediatric Quality of Life Inventory^TM^ (PedsQL 4)

Pediatric Quality of Life Inventory^TM^ (PedsQL) 4.0 Generic Core Scales is a parent proxy-report including Physical, Emotional, Social, and School Functioning Scales. It assesses parents’ perception of their child’s Health-Related Quality of Life. Higher scores represent better quality of life. It has been used frequently and is well validated within pediatric oncology populations. The PedsQL has demonstrated good psychometric properties across studies including Cronbach’s alphas that met or exceeded 0.70 and good construct validity in pediatric cancer samples (Varni et al., 1999; Racine et al., 2018). The scale has good internal consistency reliability for the total scale score (alpha = 0.90 on parent report).

### Procedure

The questionnaires were administered to the parents at the beginning of the pandemic lockdown. The researchers explained the purpose of the study to the parents.

Their written informed consent to participate in the study was obtained, and they were reassured about the confidentiality of the information they provided. Most parents preferred to be interviewed rather than to complete the questionnaires on their own. For each patient included in the study, the questionnaires were individually administered to the parents. The parents were informed that the IES-R scale referred to their child’s cancer diagnosis, while the other questionnaires referred to the current phase of the pandemic.

### Statistical Analysis

Correlations between the scores of the 4 scales were analyzed. Subsequently, the correlation between the questionnaires (IES-R, PSS, STAI-Y, PedsQL) and the variable “months,” the time between oncological diagnosis to starting the study, was determined. Comparisons between groups of parents were made using the Mann-Whitney *U* test for non-parametric samples. Statistical analysis was performed using R 4.0.3 version.

## Results

The study included the parents of 45 patients (32 with solid tumors and 13 with malignant hematological diseases). They were divided into 2 groups, those who had completed their treatment regimen (off-therapy group, *n* = 27) and those who were still receiving treatment (in-treatment group, *n* = 18). There were 18 females and 27 males.

One father had died, 1 father had psychiatric disorders, and 5 parents (1 mother and 4 fathers) did not fill in the questionnaires. Therefore, a total of 80 parents (44 mothers and 36 fathers) were included in the study. All parents agreed to participate and provided written informed consent. Demographic characteristics of the participants are summarized in Table 1.

**TABLE 1:** Demographic and clinical characteristics.

Parents (n)	80
**Patients (n)**	45
Age at diagnosis (years) mean range	7.96 ± 5.62 2–21 years
Age at study (years) mean range	13.31 ± 6.86 3–25 years
**Gender**	
Female	18
Male	27
**Cancer diagnosis**	
Leukemia	9
Lymphoma	4
Solid tumors	32
**Treatment status**	
In-treatment	18
Off-therapy	27
**Parents (n)**	80
Age at study (years) mean range	47.34 ± 6.57 30–50 years
**Relationship to patient**	
Mother	44
Father	36
**Level of schooling**	
Secondary school	29
High school and bachelor degree	36
Higher education	15
**Employment status**	
Housewife	19
Teacher	5
Employee	14
Nurse	5
Free lance	10
Worker	11
Military employee	5
Artisan	9
Unemployed	2
**Numbers of children**	
Only child	9
More than one child	36

A total of 87.5% of the parents had a moderate (*n* = 20) or high (*n* = 50) risk for traumatic disorder (IES-R, *x* = 41.68 ± 16.72), and 83.7% had a moderate (*n* = 54) or high (*n* = 13) presence of stress symptoms (PSS, mean = 19.25 ± 5.33). In our sample 75% of parents exhibited remarkable levels of anxiety, with 60 subjects in state scale and 45 subjects in trait scale having scores that reached and exceeded the STAI-Y cut off. The mean values were Y1 (state), *x* = 42.48 ± 4.32 and Y2 (trait), *x* = 41.15 ± 4.56.

**FIGURE 1:**
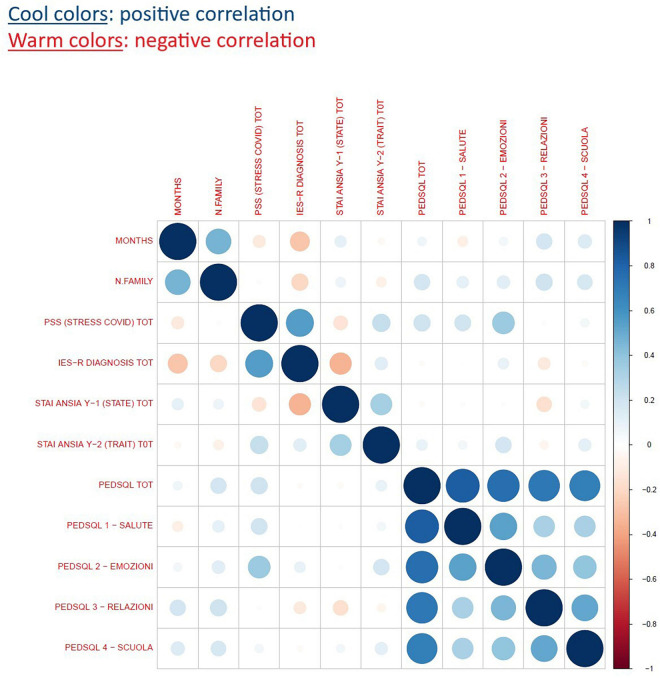
Cross correlation matrix.

The bivariate matrix of correlation (Figure 1 and Table 2) found a strong significant positive correlation between the IES-R and PSS scores (*r* = 0.55, *P* < 0.001). There was a positive correlations between the PSS and PedsQL (emotional needs) scale (*P* < 0.001) and a negative correlation between IES-R and STAI-Y (*P* < 0.001).

**TABLE 2:** Cross correlation matrix coefficients (up) and Pearson correlation tests *P*-Values (down) in each item of the table.

Correlation matrix											

	**1**	**2**	**3**	**4**	**5**	**6**	**7**	**8**	**9**	**10**	**11**
**(1) MONTHS**	1										
**(2) N. FAMILY**	0.46704 0.00001	1									
**(3) PSS (STRESS COVID) TOT**	−0.11761 0.30196	0.01631 0.88658	1								
**(4) IES-R DIAGNOSIS TOT**	−0.27920 0.01270	−0.20633 0.06810	0.55555 <0.00001	1							
**(5) STAI ANSIA Y-1 (STATE) TOT**	0.10116 0.37499	0.07998 0.48349	−0.14880 0.19060	−0.347998 0.00167	1						
**(6) STAI ANSIA Y-2 (TRAIT) T0T**	−0.03247 0.77632	−0.07877 0.49015	0.23348 0.03837	0.122036 0.28399	0.33765 0.00234	1					
**(7) PEDSQL TOT**	0.06279 0.58248	0.18741 0.09816	0.20701 0.06718	−0.020476 0.85785	−0.02569 0.82221	0.09087 0.42577	1				
**(8) PEDSQL 1** – **SALUTE**	−0.08289 0.46767	0.10616 0.35176	0.19694 0.08192	−0.006373 0.95555	0.01834 0.87255	0.05999 0.59942	0.82701 <0.00001	1			
**(9) PEDSQL 2** – **EMOZIONI**	0.05959 0.60192	0.12658 0.26631	0.36331 0.00099	0.092094 0.41954	0.01375 0.90424	0.18840 0.09636	0.75521 <0.00001	0.539688 <0.00001	1		
**(10) PEDSQL 3** – **RELAZIONI**	0.18538 0.10191	0.20458 0.07052	0.01428 0.90060	−0.112361 0.32418	−0.16864 0.13737	−0.05471 0.63205	0.71189 <0.00001	0.32832 0.00314	0.45039 0.00003	1	
**(11) PEDSQL 4** – **SCUOLA**	0.14055 0.21664	0.17065 0.13266	0.05980 0.60061	−0.028581 0.80256	0.05759 0.61413	0.11877 0.29717	0.68242 <0.00001	0.327831 <0.00001	0.39479 0.00032	0.51120 <0.00001	1

**FIGURE 2:**
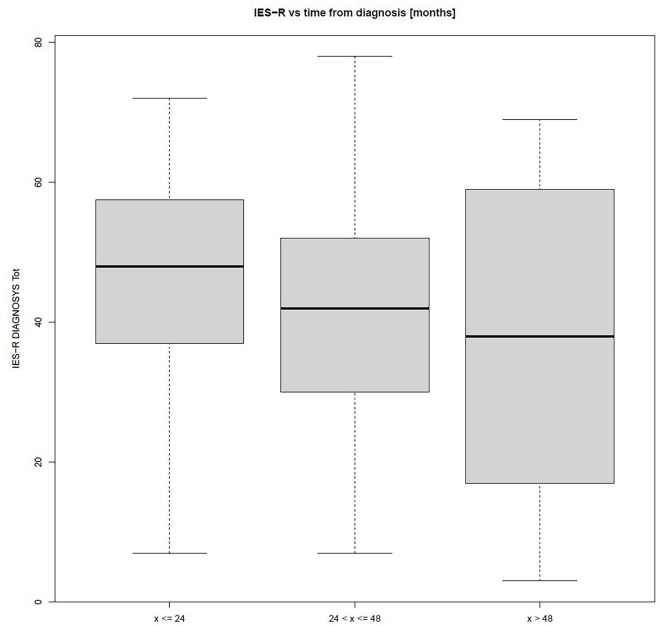
Time from diagnosis and Impact of Event Scale-Revised (IES-R) results.

It was not possible to make comparisons between the parents of patients diagnosed during and before the pandemic due to the inhomogeneity between the groups. However, the 8 parents interviewed whose children were diagnosed during the pandemic had an average IES-R of 50.28. Since there was a weak correlation (*P* > 0.05) between the time from diagnosis to completing the IES-R, the sample was further divided according to the time from diagnosis, <24, 24 – 48, and >48 months. In Figure 2 the trend line shows a decrease over time, but the difference between the groups is not significant, and in the third group (*x* > 48 months) there is also a large dispersion of scores. Comparisons between the groups were also made separating parents into those with and without high trait anxiety (divided according to the STAI-Y2 cut-off). Even this group of patients did not have significant differences in any of the test variables.

Separating the groups into those off and on therapy found that this variable had a significant impact on the outcome of IES-R (*P* < 0.001; off-therapy, *x* = 36.60 ± 4.84; on-therapy, *x* = 49.55 ± 16.23) and PSS (*P* < 0.001; off-therapy, *x* = 18.10 ± 4.84; on-therapy, *x* = 21.03 ± 5.64). Subsequent comparisons between groups found a significant difference between the scores of mothers and fathers only on the PSS (*P* < 0.001; mothers, *x* = 20.89 ± 4.90; fathers, *x* = 17.20 ± 5.21).

## Discussion

The diagnosis of cancer in the pediatric age group is widely described as a traumatic event for the parents, and may result in experiences, emotions, and even symptoms of psychopathological conditions such as post-traumatic stress disorder (Santacroce, 2002; Landolt et al., 2003; van Warmerdam et al., 2019), studies offer discordant results (Norberg and Boman, 2013; Ringnér et al., 2015). Generally, parents’ high levels of anxiety and distress following their child’s diagnosis (Patiño-Fernández et al., 2008; Vrijmoet-Wiersma et al., 2008) decrease over time with a decline already present 3 months after diagnosis (Harper et al., 2013; Scarponi et al., 2017).

The principal objective of the present study was to explore the psychological impact on parents of children with cancer during the health emergency caused by the COVID-19 pandemic. We were interested in determining if, unlike a period without a socio-sanitary emergency, the influence of post-traumatic experiences might exacerbate the challenges or symptoms, such as anxiety or stress, or place parents at a new or additional risk of psychological suffering (Evans et al., 2020). This supposition is supported by evidence showing that COVID-19 has great emotional impact, even on the general population, with or without specific medical conditions (Sani et al., 2020).

Consistent with our hypothesis, our sample parents showed high levels of post-traumatic symptoms related to the oncological diagnosis of their child, even at a time remote from diagnosis (Ringnér et al., 2015; Ribeiro da Silva, 2018). They also had an elevated perception of stress symptoms referable to the pandemic, much higher than the general population’s during COVID-19 (Limcaoco et al., 2020; Pedrozo-Pupo et al., 2020). The average level of state anxiety, measured with STAI-Y1, indicated the presence of a considerable number of anxiety symptoms.

The correlation matrix (two-tailed) showed that parents recording a higher traumatic impact level on the IES-R for child cancer diagnosis also perceived higher stress levels caused by the COVID-19 measured with the PSS. It suggests the possibility of identifying populations at risk for experiencing sequelae and consequences on child well-being. Indeed, studies have shown that parents experiencing greater stress find it more difficult to understand their child’s needs and respond in a sensitive manner (Scaramella et al., 2008; Spinelli et al., 2020) and that parenting stress might have detrimental effects on children (Giannotti et al., 2021).

The results of this study show that parents who exhibit symptoms of post-traumatic stress related to their child’s diagnosis appear to be more vulnerable to stress symptoms perceived during the pandemic lockdown. The parents’ symptom states do not appear to be related to the individual characteristics of anxiety traits. In fact, comparing the scores of “anxious” and “non-anxious” parents, there were no significant differences on all questionnaires except for the form of state STAY-Y 1.

Data from the few parents who received the oncological diagnosis of their child during the pandemic show a high level of PSS. Parents who received the diagnosis close to the onset of the COVID-19 pandemic were subjected to this potential acute stress event (Spinelli et al., 2020) and showed an increase in the already high risk (Santacroce, 2002; Norberg and Boman, 2013) of developing post-traumatic symptoms.

Therefore, it seemed worthwhile to investigate the correlation with temporal distance from the time of diagnosis to understand the role of time as a protective factor (Vrijmoet-Wiersma et al., 2008; Lazor et al., 2019). Among this study’s participants, the variable “months” from the time of diagnosis did not have a significant impact on parent score. To understand this phenomenon, it is important to consider that the scores of most parents documented a significant presence of post-traumatic symptoms. In accord with the literature, traumatic psychological conditions can have long term consequences (Porges, 2009; Kolacz et al., 2019). The COVID-19 pandemic has rapidly affected the care for children with cancer worldwide (Bouffet et al., 2020) and parent perception of assistance (Guidry et al., 2021; Mirlashari et al., 2021). Italian research also documents downstream consequences on the psychosocial functioning of tumor survivors (Fisher et al., 2021); therefore we were interested to collect parents’ impressions of their child’s quality of life (van Gorp et al., 2021). Through the inclusion of PedsQL parent proxy-report version made it possible to collect important information on the children’s activities and behaviors during COVID-19 (physical, scholastic and social activity) as well as the degree of emotional needs or difficulties of children. This last variable showed a significant positive correlation (*P* < 0.001) with the tool on parental stress (PSS), highlighting a strong relationship between the psychological state of child and parent (Kohlsdorf and Costa Junior, 2012; Salvador et al., 2019; Santos et al., 2019; Tillery et al., 2020). Previous reports have found a significant difference between mothers’ and fathers’ scores on PSS (*P* < 0.001) (Hoekstra-Weebers et al., 2001; Yeh, 2002; Norberg and Boman, 2013; Compas et al., 2015). Our results also show a significant difference between parents of patients “off therapy” and those still “on therapy” in IES-R (*P* < 0.001) and PSS (*P* < 0.001) scores. Studies show that it is important to observe the stress of parents of children with cancer throughout their lives (Ringnér et al., 2015; Ribeiro da Silva, 2018), but in this particular emergency it seems to be very important to do so during treatment, when children are most at risk of infection (Auletta et al., 2020; Bouffet et al., 2020; Evans et al., 2020; Seth, 2020; Seth et al., 2020).

## Conclusion

Worldwide, data suggests that pediatric cases of COVID-19 are less severe than adults (Evans et al., 2020). However, the possibility that their child might be infected creates worry and fear in parents, especially if the child has a pre-existing condition such as cancer where infection with COVID-19 might aggravate symptoms and pose an additional risk to the child’s health. Preliminary results of our longitudinal study, which will continue to investigate parental symptoms and variables over the course of 9 months, show a significant positive correlation between parental scores of traumatic impact of their child’s cancer diagnosis (IES-R) and parental stress perception during the COVID-19 outbreak (PSS).

The COVID-19 pandemic has introduced new challenges for the organization of health services and multidisciplinary work (Amicucci et al., 2020). This study highlights the importance of integrating care for the parents with care for the child through continuous monitoring of their psychological state and the need for parent-oriented interventions.

The present study is limited by the absence of a comparison group of parents who have not experienced the pandemic, and there is no control group of parents whose children do not have a cancer diagnosis. We will attempt to increase the reliability of the investigation by making comparisons between subjects with the re-test that will be performed, according to the study’s protocol, in the coming months.

## Data Availability Statement

The raw data supporting the conclusions of this article will be made available by the authors, without undue reservation.

## Ethics Statement

The studies involving human participants were reviewed and approved by Fondazione Policlinico Universitario Agostino Gemelli IRCCS Rome. The patients/participants provided their written informed consent to participate in this study.

## Author Contributions

AG, EM, LP, AR, and DC were involved in study planning and led to the preparation of the manuscript. AG, EM, LP, ND, GT, GA, MB, VV, and DC were involved in study conduct. All authors were involved in the reporting and reviewing of the manuscript.

## Conflict of Interest

The authors declare that the research was conducted in the absence of any commercial or financial relationships that could be construed as a potential conflict of interest.

## Publisher’s Note

All claims expressed in this article are solely those of the authors and do not necessarily represent those of their affiliated organizations, or those of the publisher, the editors and the reviewers. Any product that may be evaluated in this article, or claim that may be made by its manufacturer, is not guaranteed or endorsed by the publisher.
